# Case of Fracture of the Skull, Successfully Treated

**Published:** 1855-03

**Authors:** George Dock

**Affiliations:** Harrisburg, Pa.


*Case of Fracture of the Skull, successfully treated.* By GEORGE DOCK, M. D., M. A. N. S., etc., Harrisburg, Pa.

On the 1st of November, 1854, about nine o'clock in the morning, Singleton Welden, æt. 34 years, during a scuffle with one of his neighbors, received a blow on the head from a spade, which felled him to the ground, rendering him insensible for a few moments. He soon recovered, however, and in the course of the morning, after the immediate effects of the concussion had subsided—sickness at the stomach, &c.—he walked several squares to the office of a justice of the peace, and entered a complaint of assault and battery. He then returned home, complained about noon of sickness at stomach, vomited, and had slight pain in his head. About 4 o'clock, P. M., he was seized with convulsions, and gradually sank into a state of unconsciousness.

About 8 o'clock that evening, Dr. S. T. Charlton, his attending physician, called at my office, gave me the above account of the case, and requested me to go with him to see his patient. I went with him, and found Welden lying on his back, in an unconscious, deep comatose state; his skin cool, breathing labored and stertorous, pupils slightly dilated, forehead covered with drops of cold perspiration, pulse about 120, confined and intermitting, and occasionally there was a convulsive twitch of right arm. His feet and legs were cold. Upon examination, I found over the ridge of the left parietal bone, a puffy tumor of the integuments, about two inches in diameter, with but a slight scratch of the skin. By manipulating directly over the swollen integuments it was impossible to ascertain the condition of the bone, but by dragging the scalp to one side a little and running my finger around the margin of the swelling, I detected, very distinctly, the sharp edge of the fractured bone, and the depression of the piece to a considerable depth. The case resolved itself clearly to us as one of *concussion* with *compression*, in our opinion requiring immediate attention. Having procured my trephining instruments, I made an H incision through the integuments, (previously removing the hair,) dissected back the flaps and laid bare the bone, which was fractured in an oval shape, to the extent of about one and a half by two inches in its diameters: the piece of bone, from its upper and anterior corner, along its upper line, back and down to its posterior and lower corner, was detached and driven in, so that its outer table was on a line with the inner table of the skull. The lower line and interior portion was bent, but firm. I placed the trephine on the anterior end of the fractured piece and perforated it, but was yet unable to raise the bone with an elevator. I then took Hey's saw and sawed across the end along the line of fracture. I then sawed across the piece lengthwise at a right angle from the first, along the lower, attached side of the piece, thus detaching the fractured piece completely, the outer table of which I removed with an elevator and my thumb. This piece was fissured into three portions, which were merely held together by the periosteum—considerable blood flowing from the bone, as well as from a small branch of the middle artery of the dura mater, which latter, I was obliged to ligate, I sponged out the wound gently with a soft sponge, and proceeded to the removal of the fragments of the internal table, which were six in number and formed a piece of much larger dimensions than the outer, shelving in some distance under the outer, sound table. The dura-mater was slightly lacerated about the centre of the wound, but no cerebral hernia presented. I searched around carefully under the shelving bone with my finger, and removed all the loose bits I could find. I then dressed the wound with a little dry lint, brought the flaps together partially, and applied a compress and light bandage.

In about twenty minutes afterwards, his pulse began to improve, and his breathing became more regular and tranquil, nature expressing herself as relieved in tones that were perceptible to us, and encouraging. I directed warm applications to his feet, sinapisms to his legs, and the administration of an enema, and left him for the night, with a full knowledge of the treacherous nature of his injuries, and with but a gloomy prospect of his recovery.

On visiting him next morning—Nov. 2d—I found a gentle, but steady development of vital action, though he was still unconscious and unable to swallow even a teaspoonful of water. The enema having produced but little effect, I ordered another and more active one. His breathing was calm, his pulse 100, and he had passed his urine during the morning.

Nov. 3d. Rested quietly last night; exhibits slight consciousness this morning, swallows a little milk that I put into his mouth with a teaspoon, and turned over on his side; reaction fair, but not violent.

4th. He is still improving, opened his eyes and looked around solicitously, but cannot speak; takes milk now and then, and seems easy; as his bowels do not respond properly to the enemata, and he is now able to swallow, we gave him calomel grs. v. Enema of castor oil in the evening.

5th. He is more lively this morning, wound depurating nicely; pulse coming up in strength; sensible, and answers yes or no to questions properly; takes milk sweetened with sugar; bowels opened last night.

℞. Hyd. c. mit., grs. vi.
Ipecac. pulv., grs. ii.

M. divid. in chart. vi, one every three hours.

6th. Improving finely. We directed one powder three times a day, for a few days.

From that time forward he steadily improved without an untoward symptom. His right arm was partially paralyzed for a week or so, but then recovered. By the middle of December he was up and about his house. At the present time he is in perfect health, bodily and mentally, and working at his trade.

*Remarks.*—The points of interest in the above case are too evident to require much comment. 1st. The case appeared as one of concussion alone, and that not very serious to a casual observer. 2d. The length of time elapsing before it unfolded its compound nature, as one of concussion with *compression.* 3d. The extent of the fracture and disparity between the external and internal surfaces. 4th. The favorable recovery, without any undue inflammatory reaction of the injured brain or its membranes, followed by the kind healing of the wound, caused by the removal of so extensive a portion of bone.

The wonderful elasticity and recuperative powers of nature were well displayed in the above case; thus encouraging and lighting up the saddened heart of the practitioner in his conflict with one of the most gloomy and desperate injuries to which his fellow man is subject. Such cases teach us that Nature is the archiater after all, requiring from man but his assisting lever, when enfeebled. Even then, he must be careful what point he selects as his fulcrum, and apply his force with such nice discrimination, as not too recklessly to shatter her tottering fabric.

I must avail myself of this opportunity to acknowledge the able assistance rendered me, during the management of the above case, by Dr. S. T. Charlton, an intelligent physician of our place; and also by Mr. J. Cameron Jones, my much esteemed and attentive pupil.

*Feb. 9th*, 1855.

